# Internal medicine residents identify gaps in medical education on outpatient referrals

**DOI:** 10.1186/s12909-020-02177-3

**Published:** 2020-07-30

**Authors:** Masha J. Slavin, Mangala Rajan, Lisa M. Kern

**Affiliations:** Weill Cornell Medicine, New York, NY 10021 USA

**Keywords:** Primary care, Medical education, Ambulatory referrals

## Abstract

**Background:**

Relevant clinical information is often missing when a patient sees a specialist after being referred by another physician in the ambulatory setting. This can result in missed or delayed diagnoses, delayed treatment, unnecessary testing, and drug interactions. Residents’ attitudes toward providing clinical information at the time of referral and their perspectives toward training on referral skills are not clear. We sought to assess internal medicine residents’ attitudes toward and experiences with outpatient referrals.

**Methods:**

We conducted a cross-sectional survey in October–December 2018 of all internal medicine interns and residents affiliated with a large, urban internal medicine residency program in New York, NY. We used a novel survey instrument that included 13 questions about attitudes toward and experiences with outpatient referrals. We used descriptive statistics to characterize the results.

**Results:**

Overall, 122 of 132 residents participated (92% response rate). Respondents were approximately equally distributed across post-graduate years 1–3. Although 83% of residents reported that it is “always” important to provide the clinical reason for a referral, only 11% stated that they “always” provide a sufficient amount of clinical information for the consulting provider when making a referral. Only 9% of residents “strongly agree” that residency provides sufficient training in knowing when to refer patients, and only 8% “strongly agree” that residency provides sufficient training in what information to provide the consulting physician.

**Conclusions:**

These results suggest a substantial discrepancy between the amount of information residents believe they should provide at the time of a referral and the amount they actually provide. Many residents report not receiving adequate training during residency on when to refer patients and what clinical information to provide at the time of referral. Improvements to medical education regarding outpatient referrals are urgently needed.

## Background

Referrals from one physician to another in the ambulatory setting are routine, happening multiple times a day for any given physician. Several studies have demonstrated inadequate communication between referring primary care physicians (PCPs) and specialists [1–3]. Consequences of inadequate communication include missed or delayed diagnoses, delayed treatment, unnecessary testing, and drug interactions [2, 4]. Multiple reasons have been proposed for insufficient information being conveyed by the referring PCP to the consulting specialist, including undervaluing of and inadequate compensation for provider communication, which can be a time-consuming endeavor [4, 5], as well as lack of standardization of referral procedures [6], and inadequate appointment times and support staff [3]. We define a PCP as a physician practicing general internal medicine in the outpatient setting.

However, it is not clear how or when patterns of insufficient communication begin. There is a paucity of research evaluating the effects of internal medicine residency training on communication patterns between PCPs and specialists. In general, little attention is paid during medical training to communication between primary care providers and specialists [7, 8]. As Kessler et al. [9] emphasize, “Consultation communication is often learned by casual observation and through trial and error” [10, 11]. Few formal models have been developed for teaching consultation communication, and these have focused largely on the inpatient and emergency medicine settings [11, 12]. A limited number of residency programs appear to have formal curricula on placing ambulatory referrals [13], and have mainly been tried in pediatric training programs [14, 15].

Our objective was to assess internal medicine residents’ attitudes toward and experiences with outpatient referrals. In particular, we sought to understand the importance that trainees place on communicating patient information to specialists and the frequency with which they themselves relay information to specialists. We aimed to understand the ways in which residents most frequently communicate with consultants, their satisfaction with residency training on outpatient referrals, and whether they have witnessed harms caused by inadequate PCP-specialist communication.

## Methods

### Overview

We conducted a cross-sectional survey of internal medicine interns and residents between October and December 2018. The Institutional Review Board at Weill Cornell Medical College approved this study.

### Setting

The study took place at a large, urban academic medical center that has 3 main outpatient sites. Each resident is assigned to one site at the beginning of residency, and each resident maintains a continuity practice at that site. The majority of residents see patients at a large, hospital-based ambulatory care center. Some residents see patients at a smaller branch of that primary care practice, and some practice at a federally-qualified health center in a neighboring borough. At all three sites, residents practice alongside primary care attending physicians. The two hospital-based ambulatory care sites provided 48,078 patient visits in 2018, with 14,965 provided by residents. At these two sites, approximately 40% of patients have Medicaid, 30% have Medicare, and 30% have commercial insurance. The residents at the two sites affiliated with the academic medical center use the same electronic health record (EHR) that is also used by specialist physicians at the same academic medical center. The residents who practice at the federally-qualified health center use a separate EHR that is not linked to the academic medical center. When and how often interns and residents see outpatients varies with the year of training: during post-graduate year 2 (PGY-2), residents usually see patients for one half-day per week, whereas during PGY-1 and PGY-3, interns and residents see outpatients in concentrated blocks (2 weeks at a time, 6 times per year). In this manuscript, we collectively refer to all internal medicine house staff (both interns and residents) as residents.

### Participant sample

This study is based on a convenience sample. We invited all physicians who were in the internal medicine residency program at one academic medical center to participate (*N* = 132).

### Survey instrument

We created a novel survey instrument that consisted of 5 questions about demographic characteristics and 13 statements related to referrals. The novel survey instrument was informed by the authors’ experiences as clinicians and as educators, observing and participating in resident-led patient encounters. We pilot tested the survey instrument with a group of 3 general internists for clarity and face validity. We revised the survey instrument based on their feedback. The 5 demographic questions asked for age, gender, year of training, intended next step after residency (including fellowship training, hospital medicine, or primary care), and the ambulatory care site at which they see patients (as shown in Table 1). The 13 statements asked respondents to state how often they did specific activities (never, rarely, sometimes, often, always) or how strongly they agreed with each statement (strongly disagree, disagree, neutral, agree, strongly agree). Table 2 includes the exact wording of each statement. The first 7 questions asked about the frequency of specific behaviors related to referrals, such as how often the respondent provides sufficient clinical information when making a referral. The last 6 questions examined the degree to which residency provides training in knowing when to refer a patient and the information that should be provided to the consultant. Residents were also asked how well they thought the ambulatory referral process works for providing patients with high quality care, and whether they have observed situations in which missing clinical information led to inappropriate testing or patient harm.

**Table 1:** Participant characteristics (*N* = 122)

	Number (*N* = 122)	Percent
Age^a^		
20–29	77	63.1
30–39	43	35.2
40–49	1	0.8
Gender		
Female	54	44.3
Male	68	55.7
Level of training		
Postgraduate Year 1	42	34.4
Postgraduate Year 2	42	34.4
Postgraduate Year 3	38	31.1
Intended next step after residency		
Primary care practice	11	9.0
Hospital medicine practice	16	13.1
Medical sub-specialty fellowship	78	63.9
Other: chief	1	0.8
Not sure	16	13.1
Current practice setting^b^		
Main hospital-based practice	87	71.3
Satellite hospital-based practice	18	14.8
Community health center	16	13.1

^a^*N* = 121

^b^*N* = 121

**Table 2:** Residents’^a^ attitudes toward and practices surrounding outpatient referrals

	Never (%)	Rarely (%)	Sometimes (%)	Usually (%)	Always (%)	N
It is important to provide the clinical reason for a referral.	0 (0)	0 (0)	4 (3)	17 (14)	100 (83)	121
It is important to provide the pertinent medical history when making a referral.	0 (0)	1 (1)	13 (11)	40 (33)	68 (56)	122
I provide the clinical reason when I make a referral.	0 (0)	3 (2)	6 (5)	43 (35)	70 (57)	122
I provide the pertinent medical history when I make a referral.	0 (0)	4 (3)	25 (20)	59 (48)	34 (28)	122
When I make a referral, I provide a sufficient amount of clinical information for the consulting provider.	0 (0)	7 (6)	42 (34)	59 (48)	14 (11)	122
To make a referral, I use the electronic health record’s referral order.	0 (0)	0 (0)	0 (0)	11 (9)	110 (91)	121
In addition to using the electronic health record’s referral order, I e-mail, message, or call the consulting physician to explain the case.	59 (48)	49 (40)	12 (10)	1 (1)	1 (1)	122
	Strongly Disagree	Disagree	Neutral	Agree	Strongly Agree	
My residency provides sufficient training in knowing when to refer a patient.	0 (0)	10 (8)	29 (24)	72 (59)	11 (9)	122
My residency provides sufficient training in what information to provide the consulting physician at the time of the referral.	1 (1)	15 (12)	41 (34)	54 (45)	10 (8)	121
The referral process in the ambulatory setting works well for providing patients with high quality clinical care.	7 (6)	24 (20)	40 (33)	43 (36)	7 (6)	121
I have observed situations in which important clinical information was missing at the time that a consulting physician evaluated a patient.	4 (3)	18 (15)	47 (39)	43 (35)	10 (8)	122
I have observed situations in which missing information at the time of a consult led to repeat testing or inappropriate testing.	8 (7)	30 (25)	39 (32)	35 (29)	10 (8)	122
I have observed situations in which missing information at the time of a consult resulted in harm for the patient (including but not limited to medication errors, misdiagnosis, unnecessary testing, and other types of harm).	33 (27)	45 (37)	35 (29)	8 (7)	1 (1)	122

^a^We refer collectively to all house staff (interns and residents) as residents

### Data collection

Surveys had a short introductory paragraph, explaining that there were no right or wrong answers and that responses would be kept confidential. Paper surveys were distributed in the didactic setting by the first author (MS). Paper surveys were returned at that same didactic session into a large manila envelope, without any identifying information marked on the surveys. As surveys were collected, respondent names were checked off on a master list, so that they were not given another survey. Completion of the survey was voluntary and had no bearing on a resident’s standing within the program. MS had no supervisory role at the time of the survey collection process. Those residents who had not completed a paper survey after approximately 2 weeks of survey collection were sent an electronic version via REDCap. A reminder email was sent approximately 2 weeks later to non-respondents, and a final reminder was sent 1 week later to non-respondents. The combination of paper-based and electronic data collection was done to maximize participation. Although the primary paper-based survey was distributed during a didactic lesson, some residents would not have attended didactic lessons during the study period due to their work schedules (e.g., night shifts, intensive care unit rotations, or away on vacation). Thus, residents who were not able to respond via paper surveys received an email, with the opportunity to respond electronically. Responses from the paper surveys were manually entered and electronically-completed surveys were automatically entered into the REDCap database.

### Data analysis

We used descriptive statistics to summarize responses to the survey. Data were analyzed using SAS (version 9.4, SAS Institute, Cary, NC). Because missing data were uncommon, we used a complete case approach for analyses**.** We conducted sensitivity analyses stratified by gender and level of training.

## Results

### Response rate and respondent characteristics

We received 122 completed surveys (92% response rate). Of those, 70 were paper surveys and 52 were online surveys. An approximately equal number of participants were in each post-graduate year (Table 1). The majority (64%) intended to pursue a medical sub-specialty fellowship, 13% intended to pursue a career in hospital medicine, and 9% intended to pursue a career in primary care. The majority of residents (71%) served as primary care providers at the resident-attending practice affiliated with the academic medical center, 15% practiced at the smaller branch of that practice, and 13% practiced at the federally-qualified health center.

### Attitudes and behavior

More than three-fourths of residents (83%) reported that it is always important to provide the clinical reason for a referral; however, when asked the frequency with which they themselves provide the clinical reason when making a referral, only 57% responded “always” (Table 2). The majority of residents (56%) report that it is always important to provide the pertinent medical history when making a referral; however, when asked the frequency with which they themselves provide the pertinent medical history when making a referral, only 28% responded “always.” When residents could calibrate their behavior with their own estimation of what is “sufficient,” only 11% reported that they always provide a sufficient amount of clinical information for the consulting provider. The large majority (91%) of respondents reported that they always use the electronic health record’s referral order, while 48% reported that they never contact the consulting physician in another way (by email, message, or phone) to explain the case.

### Residency training and the referral process

Only 9% of residents strongly agree that residency provides sufficient training in knowing when to refer a patient (Table 2). Only 8% of residents strongly agree that residency provides sufficient training in what information to provide the consulting physician at the time of the referral. Six percent of residents strongly agreed that the referral process in the ambulatory setting works well for providing patients with high quality clinical care.

### Consequences

Of the sample, 8% strongly agreed and 35% agreed that they have observed situations in which important clinical information was missing at the time that a consulting physician evaluated a patient. Separately, 8% strongly agreed and 29% agreed that they had observed situations in which missing information at the time of consultation led to repeat or inappropriate testing. Lastly, 1% strongly agreed and 7% agreed that they had observed situations in which missing information at the time of consult resulted in harm for the patient.

Sensitivity analyses stratified by gender and level of training showed no difference in findings based on these demographic factors.

## Discussion

Our findings demonstrate a marked discrepancy between the amount of information that residents believe they should provide at the time of a referral and the amount that they actually provide. The large majority of residents communicate with the consultant only through the required referral order in the electronic health record, and nearly half (48%) never contact the specialist in other ways, such as by telephone, email, or messaging. Many residents feel that they have not received adequate training during residency on when to refer patients and what clinical information to convey. Nearly half (43%) have observed situations in which clinical information was missing at the time a consulting specialist evaluated a patient, one-third (37%) believe this has resulted in repeat or inappropriate testing, and nearly one-tenth (8%) have witnessed resulting harm to patients.

The Accreditation Council for Graduate Medical Education (ACGME) expects residents to communicate effectively with other physicians, work effectively within a health care team, and coordinate patient care within the health care system [16]. Obtaining these skills during residency is important, both because trainees are unlikely to receive this education after they complete their training and because residency training has a significant impact on how they will practice throughout their careers [17]. To our knowledge, this is the first study to directly measure internal medicine residents’ attitudes toward referrals, their estimates of their own behavior, and their views on the adequacy of their training in the U.S. Our findings add to the results of a physician survey done in the United Kingdom (U.K.) that also indicated that additional training on outpatient referral processes is needed [18]. A survey of 42 newly qualified physicians at a teaching hospital in the U.K. found that only 43% had received training on making referrals and 48% did not feel confident making referrals. Our findings are consistent with those of a survey of pediatric residents, in which the majority desired additional education on the referral process [15]. These results indicate an urgent need to improve resident education in this area.

Few residency programs appear to have formal training on ambulatory referrals. An evaluation of the referral curricula of 24 primary care residencies in North and South Carolina and Virginia found that fewer than one-third of programs have a formal curriculum on the process of making a referral or a curriculum on the appropriateness of subspecialty referrals [13]. In recent years, two strategies have been developed to guide emergency medicine trainees in placing consults [11, 12]. Residents trained in the 5C’s approach (contact, communicate, core question, collaboration, closing the loop) felt more confident in their communication skills and scored better on consult assessments compared to residents in the control group [19]. The PIQUED model adds a component of teaching and feedback at the end of a consult [20]. In pediatric residency training, one program implemented tools to promote discussion and feedback between residents and preceptors prompting reflection about referrals [14]. Residents noted multiple learning gains, including new medical knowledge about red flags for referral [14]. A proposed pediatric curriculum addresses the appropriateness of the referral, the logistics for completion of the referral, and appropriate post-referral care coordination and follow-up, but has not yet been rigorously evaluated [15].

Formal curricula are needed to train internal medicine residents on optimal processes for making outpatient referrals. Yet the strategies for doing so remain underdeveloped. A Cochrane review identified interventions designed to change referral rates and improve referral appropriateness but did not focus on communication patterns [21]. Forrest [8] outlines a typology of specialists’ roles to provide a framework for when patients should be referred and the subsequent coordination and co-management between the referring provider and specialist, but this conceptual approach has not been translated into a formal curriculum. Clearly, more work is needed to design, implement, evaluate and disseminate effective interventions for improving internal medicine residents’ approach to outpatient referrals.

This study has several limitations. The study was conducted at one institution, which is a large, academic medical center in an urban setting. This may limit generalizability to other settings. This survey may be subject to social desirability bias, in which respondents would give what they thought were socially desirable answers; however, such a bias would mean that our results are an underestimate of the magnitude of the problem. This survey may also be subject to recall bias; it is possible that residents may have forgotten about training that they were actually provided; however, if this were the case, the education they received did not have a lasting impact. Survey data reflected perceptions. Whether residents over- or underestimate the extent to which they document reasons for referrals is not known. Future research could compare perceptions to data collected from medical records. There may be additional reasons beyond lack of training that prevent residents from providing the information they intend to provide, such as systems-based challenges (like the need to see high volumes of patients in the ambulatory setting); future studies could inquire directly about these additional factors. This survey did not elicit information on the specific harms witnessed by residents as a result of inadequate communication. The magnitude of observed harm is sufficient that it warrants future research to better understand the details of these events and how they could be prevented in the future. Lastly, this study did not address the clinical appropriateness or indications for referral; additional research could elaborate on this topic.

This study reveals serious gaps in medical education regarding training residents on when to refer their patients to other ambulatory physicians, what clinical information should be provided at the time of the referral, and how to consistently deliver that information. That 37% of residents have observed gaps in information leading to repeat or inappropriate testing and that 8% have observed patients suffer harm from gaps in information at the time of a referral should be cause for concern. Effective interventions to address these issues are urgently needed. Improving medical education on ambulatory referrals may have both immediate and lasting effects, as residents carry their habits learned in residency into their time as attendings.

## Conclusions

This study adds new knowledge on internal medicine residents’ views of the ambulatory referral process. It demonstrates a discrepancy between the amount of information residents believe they should provide at the time of referral, and the amount they actually provide. Many residents report not receiving adequate training during residency on when to refer patients and what clinical information to provide at the time of referral. Our findings underscore a need to improve medical education on the outpatient referral process. They should inform the next steps of curriculum design and implementation of programs that will improve house officer training and care coordination between primary care providers and specialists.

## Supplementary information

**Additional file 1 Supplemental Table 1.** Residents’^1^ Attitudes Toward and Practices Surrounding Outpatient Referrals by Level of Training.

**Additional file 2 Supplemental Table 2.** Residents’^2^ Attitudes Toward and Practices Surrounding Outpatient Referrals by Gender.

## Data Availability

The datasets used and analyzed during the current study are available from the corresponding author on reasonable request.

## Abbreviations

PCP: Primary care physician
EHR: Electronic health record
PGY: Post-graduate year
ACGME: Accreditation Council for Graduate Medical Education
UK: United Kingdom
