# Supplementary material for: Internal medicine residents identify gaps in medical education on outpatient referrals
Source: BMC Med Educ. 2020 Jul 30;20:243. doi: 10.1186/s12909-020-02177-3 (PMC7392837; doi:10.1186/s12909-020-02177-3)
Supplement: Supplementary file 1 — Additional file 1 Supplemental Table 1. Residents’1 Attitudes Toward and Practices Surrounding Outpatient Referrals by Level of Training. [file 12909_2020_2177_MOESM1_ESM.docx]

**Supplemental Table 1. Residents’^[[1]](#footnote-1)^ Attitudes Toward and Practices Surrounding Outpatient Referrals by Level of Training**

| Mean Scores (1=Never to 5=Always) | PGY 1 |  | PGY 2 |  | PGY 3 |  | P for trend |
| --- | --- | --- | --- | --- | --- | --- | --- |
|  | Mean | SD | Mean | SD | Mean | SD |  |
| It is important to provide the clinical reason for a referral. | 4.81 | 0.46 | 4.90 | 0.3 | 4.65 | 0.63 | 0.16 |
| It is important to provide the pertinent medical history when making a referral. | 4.48 | 0.63 | 4.52 | 0.59 | 4.29 | 0.9 | 0.26 |
| I provide the clinical reason when I make a referral. | 4.52 | 0.59 | 4.48 | 0.71 | 4.42 | 0.83 | 0.52 |
| I provide the pertinent medical history when I make a referral. | 3.88 | 0.71 | 4.21 | 0.68 | 3.92 | 0.94 | 0.38 |
| When I make a referral, I provide a sufficient amount of clinical information for the consulting provider. | 3.67 | 0.65 | 3.71 | 0.81 | 3.58 | 0.83 | 0.15 |
| To make a referral, I use the electronic health record’s referral order. | 4.90 | 0.29 | 4.90 | 0.3 | 4.92 | 0.27 | 0.81 |
| In addition to using the electronic health record’s referral order, I e-mail, message, or call the consulting physician to explain the case. | 1.57 | 0.67 | 1.74 | 0.8 | 1.66 | 0.82 | 0.99 |
|  |  |  |  |  |  |  |  |
| My residency provides sufficient training in knowing when to refer a patient. | 3.57 | 0.63 | 3.83 | 0.82 | 3.66 | 0.78 | 0.91 |
| My residency provides sufficient training in what information to provide the consulting physician at the time of the referral. | 3.37 | 0.73 | 3.69 | 0.98 | 3.34 | 0.78 | 0.80 |
| The referral process in the ambulatory setting works well for providing patients with high quality clinical care. | 3.43 | 0.83 | 2.74 | 1.13 | 3.32 | 0.88 | 0.33 |
| I have observed situations in which important clinical information was missing at the time that a consulting physician evaluated a patient. | 3.26 | 1 | 3.04 | 0.85 | 3.63 | 0.85 | 0.06 |
| I have observed situations in which missing information at the time of a consult led to repeat testing or inappropriate testing. | 3.05 | 1.2 | 2.95 | 1.03 | 3.24 | 1 | 0.33 |
| I have observed situations in which missing information at the time of a consult resulted in harm for the patient (including but not limited to medication errors, misdiagnosis, unnecessary testing, and other types of harm). | 2.12 | 0.89 | 2.07 | 0.89 | 2.34 | 1.02 | 0.35 |

1. We refer collectively to all house staff (interns and residents) as residents [↑](#footnote-ref-1)
