# Supplementary material for: Internal medicine residents identify gaps in medical education on outpatient referrals
Source: BMC Med Educ. 2020 Jul 30;20:243. doi: 10.1186/s12909-020-02177-3 (PMC7392837; doi:10.1186/s12909-020-02177-3)
Supplement: Supplementary file 2 — Additional file 2 Supplemental Table 2. Residents’2 Attitudes Toward and Practices Surrounding Outpatient Referrals by Gender. [file 12909_2020_2177_MOESM2_ESM.docx]

**Supplemental Table 2. Residents’^[[1]](#footnote-1)^ Attitudes Toward and Practices Surrounding Outpatient Referrals by Gender**

| Mean Scores (1=Never to 5=Always) | Male |  | Female |  | Sign. |
| --- | --- | --- | --- | --- | --- |
|  | Mean | SD | Mean | SD |  |
| It is important to provide the clinical reason for a referral. | 4.73 | 0.54 | 4.87 | 0.39 | 0.26 |
| It is important to provide the pertinent medical history when making a referral. | 4.28 | 0.77 | 4.63 | 0.59 | 0.06 |
| I provide the clinical reason when I make a referral. | 4.43 | 0.8 | 4.54 | 0.57 | 0.39 |
| I provide the pertinent medical history when I make a referral. | 3.87 | 0.79 | 4.19 | 0.75 | 0.25 |
| When I make a referral, I provide a sufficient amount of clinical information for the consulting provider. | 3.5 | 0.74 | 3.85 | 0.74 | 0.11 |
| To make a referral, I use the electronic health record’s referral order. | 4.88 | 0.32 | 4.94 | 0.23 | 0.25 |
| In addition to using the electronic health record’s referral order, I e-mail, message, or call the consulting physician to explain the case. | 1.68 | 0.82 | 1.63 | 0.68 | 0.42 |
|  |  |  |  |  |  |
| My residency provides sufficient training in knowing when to refer a patient. | 3.68 | 0.7 | 3.7 | 0.82 | 0.4 |
| My residency provides sufficient training in what information to provide the consulting physician at the time of the referral. | 3.46 | 0.84 | 3.5 | 0.87 | 0.91 |
| The referral process in the ambulatory setting works well for providing patients with high quality clinical care. | 3.24 | 1.05 | 3.06 | 0.93 | 0.34 |
| I have observed situations in which important clinical information was missing at the time that a consulting physician evaluated a patient. | 3.31 | 1 | 3.3 | 0.86 | 0.79 |
| I have observed situations in which missing information at the time of a consult led to repeat testing or inappropriate testing. | 2.99 | 1.06 | 3.19 | 1.07 | 0.12 |
| I have observed situations in which missing information at the time of a consult resulted in harm for the patient (including but not limited to medication errors, misdiagnosis, unnecessary testing, and other types of harm). | 2.19 | 0.98 | 2.15 | 0.88 | 0.19 |

1. We refer collectively to all house staff (interns and residents) as residents [↑](#footnote-ref-1)
